# Will Green Finance Contribute to a Green Recovery? Evidence From Green Financial Pilot Zone in China

**DOI:** 10.3389/fpubh.2021.794195

**Published:** 2021-11-19

**Authors:** Jun Hu, Juncheng Li, Xiangyu Li, Yueyue Liu, Wenwei Wang, Liansheng Zheng

**Affiliations:** ^1^School of Management, Hainan University, Haikou, China; ^2^Institute of Finance & Banking, Chinese Academy of Social Sciences, Beijing, China; ^3^The Graduate School of College of Arts and Sciences, Georgetown University, Washington, DC, United States; ^4^School of Literature, University of Chinese Academy of Social Sciences, Beijing, China; ^5^School of Finance, Central University of Finance and Economics, Beijing, China

**Keywords:** COVID-19, green recovery, green finance, green enterprise, pilot zone, enterprise value

## Abstract

In the post-epidemic era, green finance plays a more significant role in supporting the “green recovery” of the economy, so it is necessary to evaluate the implementation effect of previous green financial policies. In 2017, the green finance reform and innovation pilot zone set up in five provinces and autonomous regions made an exploration in the development of green finance. From the perspective of micro-enterprises, can this policy play a beneficial policy effect in the long run? Based on the quasi-natural experiment of green finance pilot, using the data of A-share listed companies, this paper empirically tests the impact of pilot policies on the long-term value of green enterprises in pilot areas. It is found that, compared with non-pilot zones, the green finance pilot enables a significant increase in the Tobin Q-measured value of green enterprises in the pilot zones. Heterogeneity analysis shows that green finance pilot has a more significant impact on non-state-owned enterprises, enterprises in traditional industries, large enterprises, and enterprises in the eastern region of China. Green finance pilot zone can achieve better policy effects in areas with stronger environmental impact regulation and higher financial development levels. The mechanism test shows that the green finance pilot affects the long-term value of green enterprises through the capital market effect improving the stock trading activity of enterprises and through the real effect improving the operational efficiency and profitability of enterprises. From the perspective of micro-enterprises, this paper enriches the research on the development effect of green finance and provides theoretical support for the effect evaluation of green finance pilot policies.

## Introduction

The COVID-19 epidemic outbreak in early 2020 has made people more aware that the development of human society is always constrained by the natural environment, that economic development and growth should be well-coordinated with the protection of the natural ecological environment, and that any attempt to make the two in opposition shall be boomeranged. To prevent similar tragedies from happening again, we must promote the green and low-carbon transformation of the economy and society and enhance the sustainability of economic growth. After suffering from the impact of the COVID-19 epidemic, we must support and promote the “green recovery” of the economy, that is, in the post-epidemic era, the production and consumption return to the pre-epidemic level in terms of quantity and quality and achieve further growth while increasing the proportion of green consumption and green production, and thereby ultimately transforming the economic structure toward a green and sustainable direction.

From the perspective of economic theory, the economic recovery period after the COVID-19 epidemic impact is an excellent opportunity to promote structural transformation and “green recovery” of the economy: on the one hand, the COVID-19 epidemic impact leads to a cliff-like decline in pollution emissions in production, and a large number of backward production capacity has been naturally eliminated; on the other hand, government departments can use less green product subsidies to stimulate more green R&D investment in the context of declining production yield, thus creating more green production capacity at a lower policy cost.

As the blood of the entity economy, finance is the core of the modern economy. Therefore, finance should play an irreplaceable role in the process of economic “green recovery” in the post-epidemic era. Green finance bears the mission of providing investment and financing, project operation, risk management, and other services for economic activities supporting environmental improvement, coping with climate change, and resource conservation and efficient utilization. Because there is a limited amount of financial funds and the need for complex relief should be given priority during the epidemic crisis, green finance can help fill the financing gap faced by green investment in the process of “green recovery” in the post-epidemic era.

To give superior support to “green recovery,” green finance needs the sustenance of micro-enterprises. Only when the value of enterprises, especially those engaged in green and low-carbon related industries, improves the practice of green finance can micro-enterprises actively participate in green investment and transformation, and relevant practices can thereby be implemented stably and achieve the far-reaching effect. China's green finance started earlier. In 1995, the former State Environmental Protection Administration of China (SEPA) and the People's Bank of China respectively, issued the *Notice on Using Green Credit to Promote Environmental Protection* and the *Notice on Implementing Credit Policy and Environmental Protection*. In 2012, the former China Banking Regulatory Commission (CBRC) issued the *Guidelines for Green Credit* and gradually improved it into a statistical system for green credit. In 2015, the *Integrated* Reform Plan *for Promoting Ecological Progress* promulgated by the State Council proposed to build a green financial system. In August 2016, the People's Bank of China, the Ministry of Finance, the National Development and Reform Commission (NDRC), the former Ministry of Environmental Protection, the former China Banking Regulatory Commission (CBRC), the China Securities Regulatory Commission (CSRC) and the former China Insurance Regulatory Commission (CIRC) jointly issued the *Guidelines for Establishing the* Green *Financial* System, which constructed the top-level design of green financial development. The report of the 19th National Congress of the Communist Party of China put forward the requirement of vigorously developing green finance. Under the guidance of this spirit, the 5th Plenary Session of the 19th National Congress deliberated and adopted the *Proposal of the Central Committee of the Communist Party of China on Formulating the 14th Five-Year Plan for National Economic and Social Development and the Long-term Goals for 2035*, which made further arrangements for ecological civilization construction and green development and further emphasized the critical role of green finance in promoting the green transformation and development of economy and society. Under such a top-level design, the pilot practice at the grassroots level also serves as a magic weapon for successful reform. On June 14, 2017, the State Council executive meeting decided to build green financial reform and innovation pilot zones with different emphasis and characteristics in provinces and autonomous regions, including Zhejiang, Jiangxi, Guangdong, Guizhou, and Xinjiang, and put forward five major reform pilot tasks. At the practical level, China has carried out the construction of the green financial innovation pilot zone and continuously expanded the scope of the pilot and made a series of beneficial explorations in green financial policy and tool innovation, which laid a solid pilot foundation for promoting the further development of green finance.

With the promotion of policies and practices, green finance has gradually become a hot topic for scholars to study. Existing academic studies have achieved many research results in terms of the definition of green finance (1–5), impact effect (6), and relevant policy tools to support the development of green finance (7–11). However, the existing research focuses on the macro-impact of green finance on economic and social development and the meso-impact on industries, while there is relatively little research on the impact on micro-enterprises, which needs further exploration.

On June 14th, 2017, the State Council executive meeting decided to build green financial reform and innovation pilot zones with different emphasis and characteristics in provinces and autonomous regions, including Zhejiang, Jiangxi, Guangdong, Guizhou, and Xinjiang. Meanwhile, five major reform pilot tasks were proposed, including supporting financial institutions to set up green financial departments or green sub-branches, encouraging the development of green credit, exploring the establishment of environmental rights and interests trading markets, opening government service channels with priority for green projects and establishing green financial risk prevention mechanisms. As for the concrete situation of pilot zones, five provinces and autonomous regions actively introduced policies and measures, designed incentive mechanisms according to local conditions to promote green finance development, and achieved remarkable development efficacy. For example, Guizhou Province explores green finance supporting green agriculture and ecological environment governance; Huzhou City, Zhejiang Province, vigorously develops green credit, which accounts for 22% of the total credit balance, and improves the statistical system of green finance and starts the service of “Lvdaitong.” Jiangxi Ganjiang New District innovates various financial products and services such as credit, securities, and insurance to provide relevant financial services for investment and financing of green ecological industries. Regional Green Finance Development Index and Evaluation Report compiled by the International Institute of Green Finance, CUFE (Central University of Finance and Economics), shows that with the policy pilot promotion, the scores of the five pilot zones in green finance development and policy promotion measures are in the first echelon in China, and the relevant pilot zones have laid a solid pilot foundation for the further development of green finance.

However, what impact does this pilot policy have on micro-green enterprises? Does it bring about a long-term value promotion to green enterprises? In order to answer this question, this paper resorts to the quasi-natural experimental scenario of green finance pilot in China and the data of green enterprises in listed companies to test the impact of green finance pilot on the value of green enterprises from long-term perspectives. Firstly, this paper uses the Differences-in-Differences (DID) method to test the impact of green finance pilots on the long-term value of green enterprises measured by Tobin Q. The results of benchmark regression and a series of robustness tests show that the pilot policy of green finance has significantly improved the long-term value of green enterprises in the pilot zones. At the same time, if a region has a more vigorous intensity of environmental regulation and a higher level of financial development, green finance pilots in the region will achieve a more obvious promotion effect for the value of local green enterprises. The above results show that China's green finance pilot policy has promoted the value of green enterprises in the pilot zones in the long run, and the pilot policy has achieved specific expected outcomes. Then, this paper tests the mechanism of green finance pilot enhancing the long-term value of green enterprises. It is found that the green finance pilot not only increases the enterprise value by improving the capital market effect of stock return and trading activity of green enterprises but also improves the value of green enterprises by relieving financing constraints, increasing the technological innovation level and improving profitability. Finally, heterogeneity analysis shows that for non-state-owned enterprises, enterprises in traditional industries, large-scale enterprises, and enterprises in the eastern region, the pilot policy of green finance has a more pronounced effect on the long-term value of green enterprises.

Such a particular institutional arrangement of green finance pilot zones in some areas in China provides a rare opportunity for quasi-natural experiments to test the microeconomic consequences of the development of green finance. Meanwhile, empirical evidence from China's pilot areas will further enrich the relevant literature on the impact effect of green finance. Compared with the available literature, this paper may have the possible marginal contributions as follows: first, from the perspective of micro-enterprises, it tests the impact of green finance development on the long-term value of green enterprises, and more comprehensively describes the micro effect of green finance development. Second, a quantitative evaluation is made for the effect of green finance regional pilot policies in China. The results of this study show that China's green finance pilot improves the value of green enterprises in the pilot areas in the long term, which manifests that from the perspective of micro-enterprises, China's green finance pilot has achieved sound policy effects. Thirdly, this paper analyzes the influence mechanism of green finance development on the value of green enterprises and finds that green finance adds value to green enterprises through capital market effect and real effect.

## Literature Review and Theoretical Analysis

China's experience in the reform lies in the successful use of experimental (pilot) methods, which is also reflected in the development of green finance. On June 14th, 2017, the executive meeting of the State Council decided to build green financial reform and innovation pilot zones with different emphasis and characteristics in five provinces and autonomous regions, including Zhejiang, Jiangxi, Guangdong, Guizhou, and Xinjiang, and put forward five major reform pilot tasks, aiming at exploring replicable and scalable experiences and enriching green financial tools and policies through the green financial pilot zones. Whether the green finance development and policy promotion measures in the five pilot zones have facilitated the development of green enterprises and industries in the current period? How to objectively and comprehensively evaluate the pilot zones' experimental effect to develop green finance better is in urgent need of in-depth academic research.

In terms of the definition of green finance, there are many related concepts. However, most of them emphasize that green finance is based on innovative financial products, markets, policies, and institutions to support energy-saving and environmental protection industries and the economy (1–5). More related studies are commenced with the real effect of green finance and the supportive policies promoting the development of green finance: Alexander (7), Campiglio (8), Thoma and Hilke (9), Monnin (10), and Campigli et al. (11) discuss the supportive policies from the aspects of fiscal and taxation policies, macro-prudential supervision and bank capital supervision, and more. Through theoretical and empirical tests, Fan et al. (6) found that enterprises with higher pollution levels could obtain fewer credit resources, and their output would also decrease with the introduction of green credit policies. Some other studies have proposed to incorporate green finance factors into traditional macro models (12–14) and thereby develop dynamic stochastic general equilibrium models (15–17) and integrated assessment models (18) used for interdisciplinary studies of economy, finance, and environment.

Finance has a significant impact on the structure and development of the entity economy, and this issue has been sufficiently discussed in the classical view of financial functions (19, 20). The above studies on green finance undoubtedly further deepen and expand this subject. Currently available studies have carried out extensive discussion on the concept, development effect, and supporting policies of green finance from qualitative and quantitative perspectives. However, these studies emphasize the macro impact of green finance on economic and social development and its mesoscopic impact on the industry sector. At the same time, there are relatively few studies on the micro impact on enterprises, lacking the discussion on enterprise value. At the same time, the existing literature on the policy effect of green finance pilots is relatively insufficient, resulting in that the green finance pilot lacks rigorous academic evaluation of policy effectiveness. Based on the quasi-natural experimental scene of green finance reform and innovation pilot zone, this paper explores the impact of green finance pilots on micro-green enterprises.

From a long-term perspective, finance is the core and blood of the modern economy. For micro-enterprises, the development of finance enhances enterprises' value and development degree mainly by relieving financing constraints and improving investment efficiency, thereby improving the operational efficiency of the micro-enterprise economy. For enterprises engaged in green eco-industry and environmental protection, due to great environmental externalities in their own business and significant uncertainties in market demand, R&D of green environmental protection technology, the traditional financial business often lacks incentives and motives to support the development of the green ecological industry. Aimed at solving the “market failure” of the financial market, with the nature of public welfare finance, the concept and practice of green finance are positioned to serve the investment and financing needs of green industries and projects, support ecological environment protection, and respond to climate change risks. Therefore, green finance can support the operation of green enterprises in alleviating financing constraints, reducing transaction friction, and reducing risks, thus improving the value of green enterprises.

Specifically, green enterprises building a green production system must take green technology innovation and progress as the basic premise and need a large amount of capital investment to support it. However, the R &D and innovation of green technology are exposed to enormous uncertainties and risks, including positive environmental externalities, resulting in that the traditional financing system has an insufficient supply of financial resources for green technology innovation. Green finance opens a new path outside the traditional financial system, which increases the financing channels for green enterprises. Various types of green financial instruments and guarantee support policies also reduce the risks for enterprises in carrying out green technology innovation. With the support of green financial resources, green enterprises can enjoy tremendous success in technological innovation, thereby realizing more output of green patents and higher value of enterprises. Second, the financial system has the function of transmitting information and reducing transaction costs. For example, under the background of financial disintermediation, commercial banks hold a more critical position in the financial system because they hold a large amount of financial transaction data, which helps alleviate information asymmetry and other problems.

Similarly, the green financial system can effectively collect and process the relevant information related to green development in the market, and the financial system has a scale economy effect in the process of collecting information so that it can accurately identify potential green projects and enterprises, and provide financial resources support for the orientation of relevant targets, thus reducing the cost of green enterprises participating in financial transactions and helping to enhance enterprise value. Third, market-oriented financial transactions help to achieve effective matching of risks and benefits. For green enterprises, a significant obstacle to their financing for green technology innovation lies in more significant uncertainty and risks. In contrast, the green financial system can provide diversified financing for green enterprises, disperse related R&D risks, and provide investors with diversified investment and risk management tools, thus expanding the scale of investment in green enterprises and ultimately enhancing their value. In addition, the development of green finance may also expand the product market of green enterprises by supporting green consumption, thus enhancing the value of green enterprises.

To sum up, with the continuous promotion of green finance pilot policy, pilot zones usher in the continuous development of green finance and provide long-term financial support for green enterprises. Therefore, the impact of the green finance pilot produces long-term, lasting effects. Based on the above analysis, this paper puts forward the hypothesis **H**_**1**_:

**H**_**1**_**:** The pilot policy of green finance is conducive to enhancing the long-term value of green enterprises in the pilot zones.

According to the above analysis, green finance considers both “environment” and “finance.” Therefore, the smooth development of green finance needs the support of sound financial infrastructure and the coordination of relevant environmental policies. Suppose a region adopts relatively strict environmental regulation policies. In that case, it will help to restrain the development of enterprises in polluting industries and guide resources to flow to enterprises in green and low-carbon industries to play a synergistic effect with the pilot policies of green finance and jointly promote the development of green enterprises. At the same time, the relatively developed financial development level can also provide excellent financial infrastructure and data support for the development of green finance, thus giving full play to the pilot role of green finance. Based on this, this paper puts forward hypothesis **H**_**2**_:

**H**_**2**_**:** If a pilot zone implements more vigorous environmental regulation intensity and has a higher level of financial development, the green financial pilot will achieve a more significant policy effect.

Studies on the channels and mechanisms that affect enterprise value primarily focus on the capital market and entities. For example, in terms of the studies on the influence of patents on the enterprise value, Long (21), Levitas and Mcfadyen (22) all maintain that patents can improve enterprise value not only by enhancing enterprise profitability but also by the channel of transmitting signals to the capital market and rising stock prices. Kruger (23), when studying the influence mechanism of carbon emission information disclosure on enterprise value, also thinks that there are capital market effects (CME) that affect the long-term trading activity of enterprise capital market and real effects (RE) that affect the operation of enterprise entities. With regards to the green finance pilot policy studied in this paper, on the one hand, the green finance pilot can convey signals of supporting the long-term development of green enterprises in the pilot zone to the capital market to reduce information friction and affect the capital market's judgment on the future business operating environment and performance of green enterprises. Thereby, green enterprises can obtain a higher market attention index (MAI) and greater market liquidity in the capital market, thus affecting enterprise value, which is the CME capital market effects channel of green finance pilot affecting the value of green enterprises. On the other hand, it is the channel of real effects (RE), that is, green finance pilot can alleviate the financing constraints faced by green enterprises to improve the investment scale and innovation R&D input and output level of green enterprises, improve the investment efficiency, reduce the agency cost, to enhance the profitability of enterprises and ultimately elevate the value of enterprises. Based on the above analysis, this paper proposes hypothesis **H**_**3**_:

**H**_**3**_**:** The pilot policy of green finance affects the long-term value of enterprises through two mechanisms: capital market effects and real effects.

## Sample Selection and Empirical Model

### Sample Selection

This paper mainly aims to test the impact of the green financial reform and innovation pilot area established in 2017 on the value of green enterprises in the pilot zones. Based on the consideration of data availability, this paper takes the green enterprises in A-share listed companies from 2014 to 2019 as the research object. In this paper, “green enterprise” is defined in benchmark regression by manually matching the main business disclosed in the enterprise's annual report with the green industries listed in the Green Industry Guidance Catalog (2019 Edition) issued by the National Development and Reform Commission. If the enterprise's primary business is included in the Green Industry Guidance Catalog, it will be regarded as a “green enterprise.” Green Industry Guidance Catalog classifies green industries into energy-saving, environmental protection industries, clean production industry, clean energy industry, eco-environmental industry, green upgrading of infrastructure, and green services, with each industry contains specific industries sub-items. In the part of robustness test, this paper further takes the pollution intensity of the industry in which the enterprise is located and the social responsibility score of the enterprise as the standard to classify “green enterprises.” For the measurement of enterprise value, this paper uses Tobin *Q* to measure the long-term value of the enterprise (23). There are several reasons why Tobin *Q* is used to measure the value of an enterprise: First, from the definition of Tobin *Q*, the concept of Tobin *Q* covers two aspects of capital market valuation and physical investment. It can realize the organic combination of capital market and real industry. The impact of the green finance pilot zone covers the above two aspects. Therefore, using Tobin's *Q* to measure corporate value can better reflect the policy effects of the green finance pilot, and it is consistent with the mechanism analysis of this paper. Second, with the increase in Tobin's *Q* value, the company's capital market valuation is gradually higher than the company's replacement cost, which will encourage companies to increase investment expenditures. The research theme of this paper is how green finance can promote green recovery. From a micro level, the green recovery of the economy after the epidemic will inevitably require green companies to expand their production and investment scale. Therefore, using Tobin's *Q* to measure corporate value can reflect the incentive effect of green finance pilots on green corporate investment, and thus better fit the research theme of this paper. The relevant data are from different sources. The relevant data are sourced from the WIND database and RESSET database, respectively. The control variables selected in this paper include enterprise development ability, enterprise price-earnings ratio, cash flow, asset size, book-to-market ratio, ROA, leverage ratio, and sales revenue growth rate, etc. The relevant data comes from the iFind database of Hithink RoyalFlush Information Network Co., Ltd.

### Model Setting and Descriptive Statistics

In this paper, a DID model is established as shown in the formula (1):


(1) $$\begin{array}{l} q_{it}\;=\;\alpha_{0}\;+\;\beta_{1}{Treat}_{i}\;\times \;{Time}_{t}\;+\;\alpha_{i}\sum {Control}_{i}\;+\;\mu_{i}\\ \;+\;{Year}_{t}\;+\;\epsilon_{it} \end{array}$$

In which: *q*_*it*_ stands for Tobin Q of the enterprise, and the dummy variable *Treat*_*i*_ has a value of 1 for the processing group and 0 for the control group; In this paper, 2014–2019 is selected as the sample interval, and 2017 is taken as the base year. The dummy variable *Time*_*t*_ takes the value of 1 after introducing the pilot policy (2017–2019); otherwise, it is 0. The main observation variable is coefficient β_1_ of interactive item *Treat*_*i*_ × *Time*_*t*_, which reflects the impact of green finance pilot policy on enterprise value. ∑*Control*_*i*_ is a group of control variables, including P/E ratio (*PE*), relative Cash flow (*Cash*), return on assets (*ROA*), leverage ratio (*lev*), sales revenue growth rate (*sales* < *uscore* > *growth*), ownership concentration (*con*), assets scale (*lnassets*), enterprise development ability (*DA*expressed as the growth rate of business revenue) and enterprise's book-to-market(*BM*). μ_*i*_ represents the fixed effect of individual enterprises, *Year*_*t*_ represents the fixed effect of years and ϵ_*it*_ is the stochastic error term. The definition and descriptive statistics of long-term value research-related variables are shown in Table 1.

**Table 1:** Descriptive statistics.

**Variable symbol**	**Meaning of variable**	**Mean value**	**Standard deviation**	**Minimum value**	**Maximum value**	**Observed value**
*q*	Enterprise value	1.706	0.733	−0.824	9.017	6,771
*PE*	Enterprise P/E ratio	2.065	2.271	0	7.032	6,771
*Cash*	Cash flow of enterprise	2.655	0.664	0.047	4.518	6,771
*ROA*	Return on assets (%)	5.445	12.612	−183.981	727.529	6,771
*Lev*	Financial leverage ratio	3.316	3.773	0.418	119.630	6,771
*Sales_growth*	Revenue growth rate (%)	18.897	67.428	−97.390	3,137.895	6,771
*Con*	Ownership concentration	55.429	17.049	0	98.585	6,771
*Lnassets*	Enterprise's assets scale	22.252	1.360	17.804	28.636	6,771
*DA*	Development ability	15.985	37.553	−60.732	214.076	6,771
*BM*	Book-to-market	0.182	0.207	0	0.857	6,771

## Empirical Results and Analysis

### Benchmark Regression Results

For the long-term effect of the green finance pilot, the results of benchmark regression are shown in Table 2. Among them, Column (1) is the regression result without added control variables, Column (2) is added with enterprise-level financial data as control variables, Column (3) is only added with enterprise capital market and governance data as control variables, Column (4) is added with various control variables, and the benchmark regression is clustered to the individual level of enterprises. From the regression results in Table 2, it can be seen that the coefficients of the core explanatory variables of benchmark regression are all significantly positive, indicating that the green finance pilot policy has a significant role in promoting and enhancing the long-term value of green enterprises measured by Tobin Q. It is proved that for green enterprises, the pilot policy of green finance enhances the long-term value of enterprises. Thus, it belongs to a “lining project” with a practical development effect.

**Table 2:** Results of benchmark regression.

	***q***	***q***	***q***	***q***
	**(1)**	**(2)**	**(3)**	**(4)**
*Treat*_*i*_×*Time*_*t*_	0.070^**^ **(2.02)**	0.065^*^ **(1.92)**	0.088^***^ **(2.95)**	0.085^***^ **(2.91)**
*Cash*		−0.104^***^ (−4.48)		−0.155^***^ (−7.53)
*ROA*		−0.003 (−1.38)		−0.002 (−1.44)
*Lev*		−0.011^***^ (−2.79)		−0.021^***^ (−3.20)
*Sales_growth*		0.000 (0.90)		0.000 (1.02)
*Lnassets*		−0.048 (−1.28)		−0.207^***^ (−5.60)
*DA*		0.000^***^ (2.14)		0.000 (1.28)
*BM*			−0.636^***^ (−11.44)	−0.572^***^ (−10.52)
*PE*			0.033^***^ (5.90)	0.029^***^ (5.52)
*Con*			0.018^***^ (17.83)	0.020^***^ (24.21)
*_Cons*	1.635^***^ (94.41)	3.014^***^ (3.68)	0.821^***^ (15.08)	5.689^***^ (7.07)
Fixed effect of business entity	Yes	Yes	Yes	Yes
Fixed effect of time	Yes	Yes	Yes	Yes
*N*	6,771	6,771	6,771	6,771
*R* ^2^	0.168	0.186	0.334	0.378

***, **, * *Represent the 1, 5, and 10% levels of significance, respectively. The values reported in brackets are t-statistics. The bold values mean regression coefficients*.

### Dynamic Effect Analysis

The difference-in-differences model aims to evaluate the policy effect through the quasi-natural experiment of public policy, so it must satisfy the parallel trend hypothesis, i.e., without policy intervention (before the pilot policy), the development trend of the explained variables in the treatment group and the control group is consistent. Concerning relevant literature, this paper constructs the following model (2) to test the dynamic effect of green finance pilot policy:


(2) $$\begin{array}{l} q_{it}\;=\;\alpha_{0}\;+\;\beta_{i}\sum_{t=2014}^{2019}year\text{\_}t\;+\;\gamma_{i}\sum {Control}_{i}\;+\;\epsilon_{it} \end{array}$$

**Figure 1:**
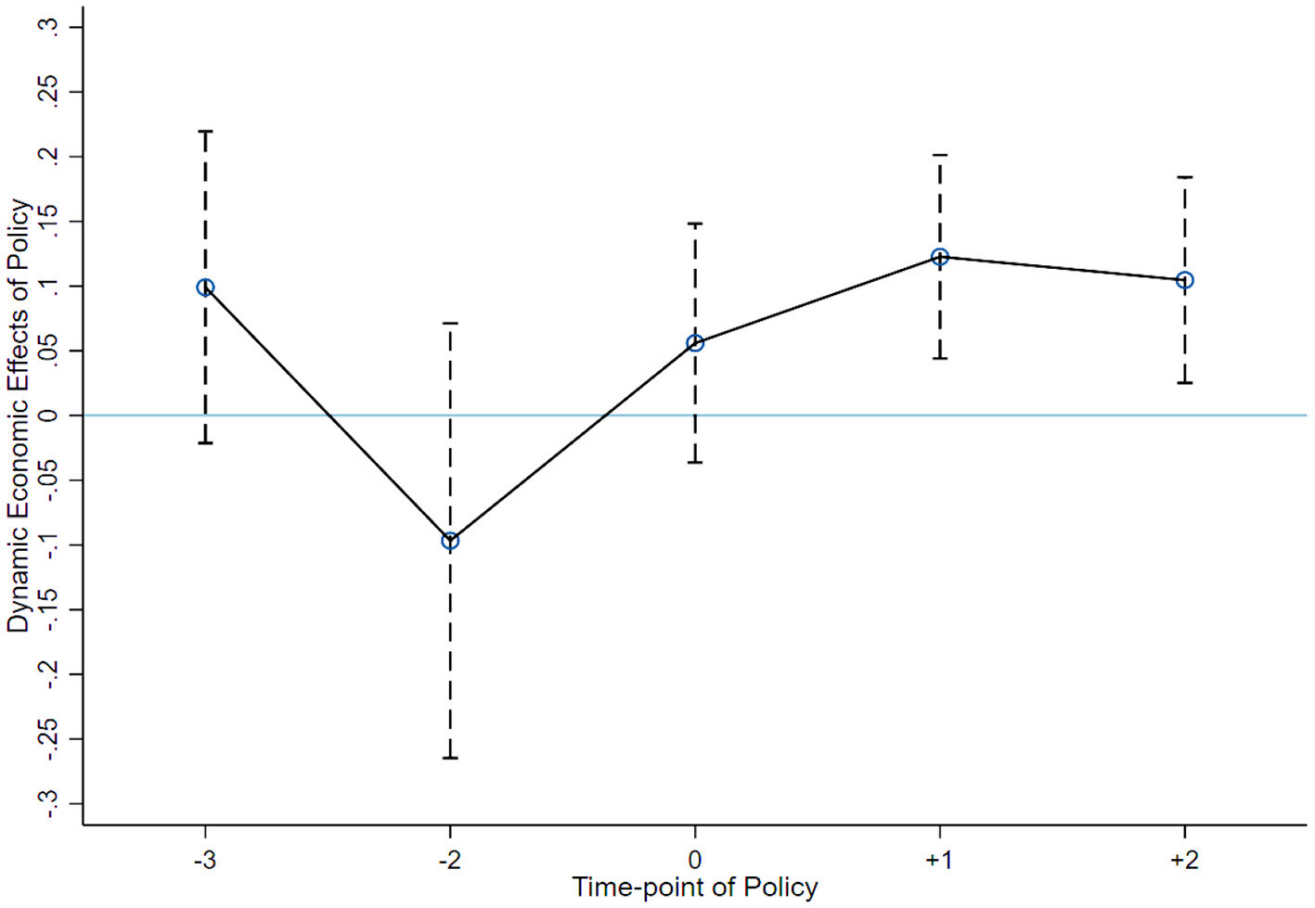
Parallel Trend.

In which, *year*_*t* = *Treat* × *year* is the product of the dummy variable of the pilot area and the dummy variable of time, and *year* is the dummy variable of time. Figure 1 shows the results of the parallel trend test, in which the year before the introduction of the pilot policy is taken as the base period. Before the pilot policy and when the policy is implemented, the coefficient of the time dynamic cross-product term is not significant, which indicates that there is no significant difference between the treatment group and the control group before the pilot policy. Hence, the parallel trend hypothesis is satisfied. In the 2 years (2018 and 2019) after the implementation of the policy, the corresponding coefficient is significantly positive, so it meets the relevant requirements of the parallel trend test.

### Robustness Test

To ensure the robustness of the regression results of the above-mentioned long-term value benchmarks of green enterprises, this paper has carried out robustness tests in the following aspects:

(1) Replace the definition criteria of “green enterprise.” Benchmark regression in this paper manually matches the main business of an enterprise with the green industries listed in the *Green Industry Guidance Catalog* (2019 Edition) issued by the National Development and Reform Commission [F.G.H.Zi (2019) No.293]. Suppose the main business is included in the Green Industry Guidance Catalog. In that case, it will be regarded as a “green enterprise.” Furthermore, this paper replaces the definition of “green enterprise.” It uses industrial pollution intensity and corporate social responsibility score respectively as the criteria to delimit “green enterprise.”

As for the industrial pollution intensity, this paper selects six industrial pollution discharges, including sulfur dioxide, smoke dust, nitrogen oxide, chemical oxygen demand (wastewater), ammonia nitrogen, and solid waste. Firstly, calculate the discharge of pollutants per unit output value of the industry, i.e., *UE*_*ij*_ = *E*_*ij*_/*O*_*i*_, in which, *E*_*ij*_ is the emission of major pollutant *j* in the industry, and *O*_*i*_ is the total output value of the industry *i*. Then, standardize each industrial sector's maximum and minimum pollutant emissions per unit output value: $UE_{ij}^{s}=\left[UE_{ij}-\min\left(UE_{j}\right)\right]/\left[\max\left(UE_{j}\right)-\min\left(UE_{j}\right)\right]$. Finally, the emissions per unit output value of various pollutants are summed up, and the obtained industrial pollution emission intensity is $\gamma_{i}=\overset{n}{\underset{j=\;1}{\sum }}UE_{ij}^{s}$.

After calculating the industrial pollution emission intensity, the industrial departments are classified according to the median. If the pollution intensity is lower than the median, the enterprises in the industry are regarded as “green enterprises.” Otherwise, they are regarded as “non-green enterprises.” The data of pollution emission and the output value of industrial sectors in this paper come from China Industry Statistical Yearbook.

For the corporate social responsibility score, this paper uses the social responsibility index of listed companies compiled by hexun.com to calculate its average score over the years in the sample period and classifies enterprises according to its median. If the average score of corporate social responsibility is higher than the median, it shows that it performs well in environmental protection and pollution reduction, so it is defined as a “green enterprise”; otherwise, it is regarded as a “non-green enterprise.”

The regression results after replacing the definition standard of “green enterprise” are shown in column (1) and column (2) of Table 3. The regression results of “green enterprises” defined by industry pollution emission intensity are reported in column (1) and the regression results of “green enterprises” defined by social responsibility score are reported in column (2). It can be seen from Table 3 that the coefficient of core explanatory variables remains positive at the significance level of 1%, which shows that after changing the definition standard of green enterprises, the corresponding regression results are still consistent with the results of benchmark regression.

**Figure 2:**
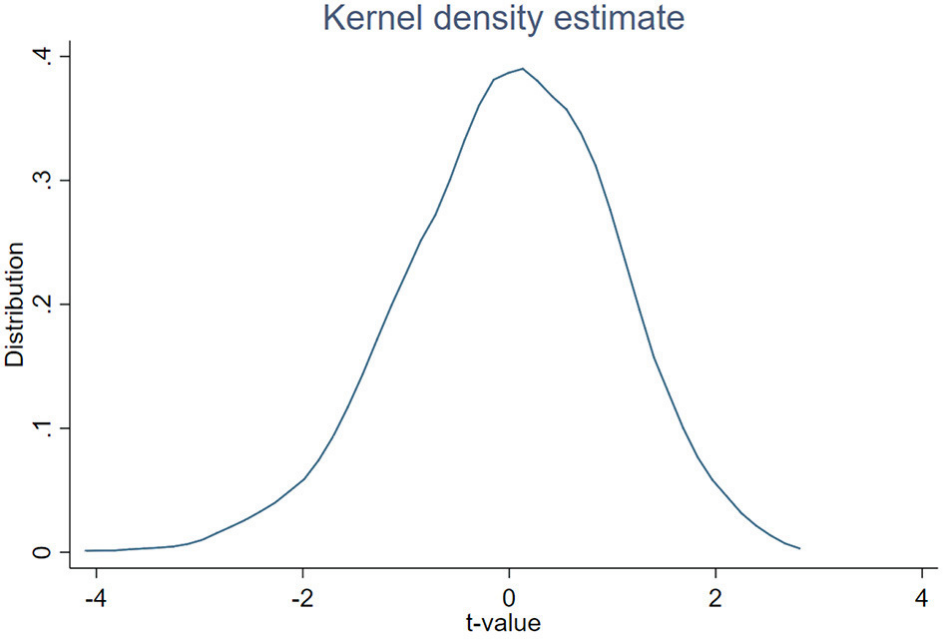
Results of Placebo Test. kernal = epanechnikov, bandwidth = 0.2256.

(2) Placebo Test. In order to further exclude the influence of other unknown factors on the selection of pilot areas and ensure that the green finance pilot causes the conclusions obtained in this paper, this paper further carries out the placebo test. Specifically, this paper takes 1,000 samples from 31 provincial administrative regions, randomly selects five provincial administrative regions as the virtual experimental group, the remaining administrative regions as the virtual control group, and carries out regression according to the benchmark model. The corresponding results are shown in Figure 2, where the horizontal axis is the *t*-value of the estimated coefficient, and the vertical axis is the corresponding distribution. It can be seen from Figure 2 that the absolute values of t values of most sampling estimation coefficients are all within 2, far less than the value *t* of the benchmark regression in this paper, and value *p* is above 0. It shows that random sampling regression has not achieved significant regression results. Therefore, the conclusion of benchmark regression in this paper has passed the placebo test, and it further proves the robustness of benchmark regression results.
(3) Adding the control variables at the regional level. In this paper, the control variables selected by the benchmark regression are all variables at the company level, and the macroeconomic variables of the company's location will also impact the enterprise value. Therefore, this paper further incorporates the indicators that measure the economic development level of each region, including GDP, per capita GDP, fiscal expenditure, total import and export volume, and the proportion of secondary and tertiary production into the control variables. The corresponding regression results are shown in column (3) of Table 3, and the regression results are still consistent with the benchmark regression.
(4) Change the standard clustering error. In this paper, the benchmark regression clustering is to the individual enterprise level, and then clustering standards are set to the regional and industry levels, respectively. The corresponding regression results are shown in columns (4) and (5) of Table 3. Column (4) reports the regression results of clustering the benchmark regression to the regional level, while column (5) clusters to the industry level. It shows that the corresponding regression results are consistent with the benchmark regression after changing the clustering criteria.
(5) PSM-DID Test. To further test the robustness of the benchmark regression of the first group of control experiments, this paper uses the tendency score matching method to match the characteristic variables of the enterprises in the control group and those in the control group to overcome the influence of sample selection bias and then carries out PSM-DID test. The corresponding regression results are shown in column (6) of Table 3. The regression results are consistent with the benchmark regression results, which is in line with expectations.
(6) Change control group and experimental group. In order to more fully explain green pilot financial policies for the influence of the green enterprise value, this paper further changes in the control group and experimental group, green businesses within the pilot provinces as the treatment group, with the non-green enterprise within the pilot provinces and regions as the control group, to compare the pilot areas within a green and not green enterprises in long-term value differences. Thus, it illustrates the influence of green finance pilot policy on the long-term relative value of green enterprises. The corresponding regression results are shown in column (7) of Table 3. It can be seen from Table 3 that compared with the non-green enterprises in the pilot provinces, the green finance pilot policy has significantly enhanced the value of green enterprises in the pilot provinces. This result further supports the conclusion drawn from the benchmark regression results that the green finance pilot has significantly enhanced the value of green enterprises from the perspective of relative value.

**Table 3:** Robustness test.

	***q***	***q***	***q***	***q***	***q***	***q***	***q***
	**(1)**	**(2)**	**(3)**	**(4)**	**(5)**	**(6)**	**(7)**
*Treat*_*i*_×*Time*_*t*_	0.101^***^ **(2.96)**	0.081^***^ **(2.64)**	0.092^***^ **(2.59)**	0.085^***^ **(3.41)**	0.085^***^ **(4.92)**	0.081^**^ **(2.27)**	0.061^*^ **(1.72)**
Control variable	Controlled	Controlled	Controlled	Controlled	Controlled	Controlled	Controlled
Entity fixed effects	Yes	Yes	Yes	Yes	Yes	Yes	Yes
Time fixed effect	Yes	Yes	Yes	Yes	Yes	Yes	Yes
*N*	7,692	9,656	6,771	6,771	6,771	4,092	4,556
*R* ^2^	0.328	0.350	0.380	0.378	0.378	0.307	0.289

***, **, * *represent the 1%, 5%, and 10% levels of significance, respectively. The bold values mean regression coefficients*.

### Analysis of Regulatory Effect

This paper further analyzes the regulatory effect of the related factors that may affect the benchmark regression conclusion. Since green finance has two significant factors, namely “environment” and “finance,” this paper focuses on the influence of regional environmental regulation and financial development level. The greater the environmental regulation in a region, the higher the environmental compliance costs faced by enterprises in the region, eventually reducing the production resources and market share allocated to polluting enterprises. The green enterprises will be able to obtain more abundant resources accordingly. Therefore, the intensity of environmental regulation and green finance pilot policy have a synergistic effect in theory, which can jointly promote the improvement of green enterprise value. For the level of financial development, if a region has a high level of financial development, it has an excellent financial development foundation to carry out green finance pilot in this region, better to play the role of the existing financial infrastructure, more accurately meet the financing needs of green enterprises, and promote the value of green enterprises. In order to test whether the above synergistic effect exists, the following interaction model is constructed in this paper (3):


(3) $$\begin{array}{l} q_{it}\;=\;\alpha_{0}\;+\;\beta_{1}Treat_{i}\times Time_{t}{}^{*}AJ_{it}\;+\;\beta_{2}Treat_{i}\times Time_{t}\\ \;+\;\beta_{3}AJ_{it}\;+\;\alpha_{i}\sum Control_{i}\;+\;\epsilon_{it} \end{array}$$

In which, *AJ*_*it*_ is the regulated variable, including the environmental regulation intensity *ER* and financial development level *Fin* of a region. The environmental regulation intensity is measured by the number of environmental penalties that have been standardized by the maximum and minimum over the years in the province where the enterprise is located, while the ratio of loans to GDP measures the level of financial development.

(1) Environmental regulation intensity of region. The regression results of the regulatory effect of environmental regulation intensity are shown in column (1) of Table 4. It can be seen from the table that the coefficient of a cross-product term is positive at the significance level of 10%, indicating that environmental regulation intensity has a positive moderating effect on green finance pilot policies. The greater the intensity of environmental regulation in a region, the more significant the promotion effect of green finance pilots on the value of green enterprises. The empirical results of this moderating effect are consistent with the above theoretical analysis.
(2) Financial development level. The regression results of the regulatory effect of regional financial development level are shown in column (2) of Table 4. It can be seen from the table that the coefficient of a cross-product term is positive at the significance level of 5%, indicating that the financial development level of a region has a positive moderating effect on green finance pilot policies. The higher a region's financial development level is, the more significant it is to promote the value of green enterprises by launching green financial pilot projects in the region. The empirical results of this regulatory effect are consistent with the above theoretical analysis.

**Table 4:** Analysis of regulatory effect.

	***q***	***q***
	**(1)**	**(2)**
*Treat*_*i*_×*Time*_*t*_×*ER*	0.128^*^ **(1.89)**	
*Treat*_*i*_×*Time*_*t*_×*Fin*		0.102^**^ **(2.02)**
*Treat*_*i*_×*Time*_*t*_	0.030 (1.44)	−0.001 (−0.03)
*ER*	−0.050 (−1.64)	
*Fin*		−0.028 (−1.37)
Control variables	Controlled	Controlled
Entity fixed effects	Yes	Yes
Time fixed effects	Yes	Yes
*N*	6,771	6,771
*R* ^2^	0.379	0.233

***, **, * *represent the 1%, 5%, and 10% levels of significance, respectively. The bold values mean regression coefficients*.

In conclusion, the stronger the environmental regulation and the higher the financial development level in a region, the more significant the policy effect of carrying out a green financial pilot in the region to enhance the value of green enterprises.

### Mechanism Test

According to the analysis of the theoretical part of this paper, the pilot policy of green finance mainly affects the value of green enterprises through two types of channels: on the one hand, it is the capital market channel, which can send the signal of supporting the development of green enterprises to the capital market, reduce the information friction, and affect the judgment of the capital market on the future business environment and performance of green enterprises, thus enabling green enterprises to gain higher market attention and greater market liquidity in the capital market, thus affecting the enterprise value; on the other hand, it is the real effect channel, that is, the green finance pilot improves the technological innovation level of green enterprises by alleviating the financing constraints faced by green enterprises, thus enhancing their profitability and ultimately enhancing their value. For this reason, this paper examines the mechanism from the above two aspects.

(1) The effect of capital market. This paper comprehensively uses the practices of relevant literature (23, 24) as a reference, uses annual stock return rate and transaction size to measure the capital market effect of green finance pilot, and constructs the following empirical model (4):

(4) $$\begin{array}{l} {CME}_{it}\;=\;\alpha_{0}\;+\;\beta_{1}{Treat}_{i}\;\times \;{Time}_{t}\;+\;\alpha_{i}\sum {Control}_{i}\;+\;\epsilon_{it} \end{array}$$

*CME*_*it*_ is the explained variable, including annual stock return rate (*yrr*) and trading size adjusted by market value (*tv*). The core explanatory variable is *did*, it mainly focuses on the coefficient β_1_ and reflects the impact of green finance pilot policies on relevant variables.

The corresponding regression results are shown in Table 5. It can be seen from Table 5 that the green finance pilot policy has significantly improved the annual rate of return and trading scale of green enterprise stocks in the pilot area. The regression results show that the pilot policy of green finance has significantly improved the active degree of trading in the capital market of green enterprises in the pilot areas, made the stocks of green enterprises in the pilot areas more popular in the capital market, and significantly improved their annual rate of return, which ultimately made the value of green enterprises in the pilot areas higher.

(2) Real effect. According to the above theoretical analysis, the green finance pilot project alleviates the financing constraints of green enterprises in the pilot area, reduces information asymmetry, reduces financing costs, and then increases their R&D investment and investment scale, realizing green technology innovation and improving the profitability of enterprises. Therefore, this paper uses SA index (*sa*) (25), the logarithm of the number of technology patents held (*patents*), and the net profit rate of sales(*sales_rate*) that indicates the profitability of enterprises to test the real effect of the green finance pilot policy. This paper constructs the following empirical model (5):

(5) $$\begin{array}{l} {RE}_{it}\;=\;\alpha_{0}\;+\;\beta_{1}{Treat}_{i}\;\times \;{Time}_{t}\;+\;\alpha_{i}\sum {Control}_{i}\;+\;\epsilon_{it} \end{array}$$

**Table 5:** Mechanism test—capital market effect.

	***yrr***	***tv***
	**(1)**	**(2)**
*Treat*_*i*_×*Time*_*t*_	0.024^*^ **(1.69)**	0.021^*^ **(1.89)**
Control variables	Controlled	Controlled
Entity fixed effects	Yes	Yes
Time fixed effects	Yes	Yes
*N*	6,567	6,554
*R* ^2^	0.322	0.206

***, **, * *represent the 1%, 5%, and 10% levels of significance, respectively. The bold values mean regression coefficients*.

In which, *RE*_*it*_ is the explained variable, including the above indicators to measure the real effect. The core explanatory variable is *Treat*_*i*_×*Time*_*t*_, it mainly focuses on the coefficient β_1_ and reflects the impact of green finance pilot policies on relevant variables.

The corresponding regression results are shown in Table 6. Table 6 shows that green pilot financial policy significantly reduced the pilot areas green enterprise financing constraints faced by index, pilot areas significantly increased investment scale as well as research and development of the green enterprise of input and output, and reduced the pilot areas green enterprise financing facing premium level, improve the profitability of the green enterprise's pilot areas. The regression results show that the pilot green finance policy for the pilot area green enterprise has a promoting effect on the level of essence, relieve the pilot areas of green enterprise financing constraints, promote the scale of the input and output of technology innovation, and reduce the financing premiums, and ultimately increase the value of the pilot area of green enterprise.

**Table 6:** Mechanism test—real effect.

	***Sa***	***Patents***	***Sales_rate***
	**(1)**	**(2)**	**(3)**
*Treat*_*i*_×*Time*_*t*_	−0.006^*^ **(−1.77)**	0.118^*^ **(1.90)**	0.018^*^ **(1.93)**
Control variables	Controlled	Controlled	Controlled
Entity fixed effects	Yes	Yes	Yes
Time fixed effects	Yes	Yes	Yes
*N*	5,650	3,287	6,742
*R* ^2^	0.804	0.052	0.145

***, **, * *represent the 1%, 5%, and 10% levels of significance, respectively. The bold values mean regression coefficients*.

In conclusion, the pilot policy of green finance enhances the attractiveness of green enterprises in the pilot area in the capital market through the channels of capital market effect and real effect, and reduces the financing constraints faced by green enterprises at the entity level, improves the input and output of innovation and research and development, reduces the level of financing premium and improves profitability, thus ultimately promoting the value of green enterprises in the pilot area.

### Heterogeneity Analysis

For green enterprises with different characteristics and regions, green finance pilot policies may have heterogeneous impacts. Therefore, this paper further conducts a heterogeneity analysis based on different characteristics of enterprises.

(1) Heterogeneity analysis based on enterprise ownership. According to the nature of ownership, enterprises can be divided into state-owned enterprises and non-state-owned enterprises. Since there are significant differences between state-owned enterprises and non-state-owned enterprises in terms of R&D resources, contact with government departments, and financing constraints, the ownership nature of enterprises may affect the role of green finance pilot in enterprise value. Compared with non-state-owned enterprises, state-owned enterprises face relatively lower financing constraints. Green enterprises in state-owned enterprises can meet their financing needs through various forms even without developing green finance, while green enterprises in non-state-owned enterprises often face considerable financing constraints. Therefore, through the pilot of green finance, the financing constraints faced by green enterprises in non-state-owned enterprises have been marginally improved to a greater extent, making the impact of the pilot policies on their enterprise value more significant. From the perspective of technological innovation, there are some differences in technological innovation efficiency between state-owned enterprises and non-state-owned enterprises: Wei et al. (26) found that the R&D efficiency of state-owned enterprises is lower than that of private enterprises on the premise of the same scale and resource mismatch. From the above mechanism analysis, it can be seen that the green financial pilot is helpful to promote the technological innovation of green enterprises. As the technological innovation efficiency of non-state-owned enterprises is higher, the expected result is that the green financial pilot has a more noticeable effect on promoting the technological innovation of non-state-owned enterprises, thus significantly enhancing the value of green enterprises in non-state-owned enterprises.

Columns (1) and (2) of Table 7 report the grouped regression results of state-owned and non-state-owned enterprises. It can be seen from Table 7 that the coefficients of core explanatory variables of both state-owned enterprises and non-state-owned enterprises are positive, but state-owned enterprises fail to pass the significance test. This result indicates that the effect of green finance pilot on enhancing the value of green non-state-owned enterprises is more significant.

**Table 7:** Heterogeneity analysis results 1.

	**State-owned enterprise**	**Non state-owned enterprises**	**Enterprise of high-tech industry**	**Enterprises in traditional industries**
	***q***	***q***	***q***	***q***
	**(1)**	**(2)**	**(3)**	**(4)**
*Treat*_*i*_×*Time*_*t*_	**0.055** **(1.23)**	0.064^*^ **(1.72)**	0.066^*^ **(1.71)**	0.085^**^ **(1.98)**
Control variables	Controlled	Controlled	Controlled	Controlled
Entity fixed effects	Yes	Yes	Yes	Yes
Time fixed effects	Yes	Yes	Yes	Yes
*N*	2,620	4,145	3,281	3,484
*R* ^2^	0.332	0.417	0.500	0.289

***, **, * *represent the 1%, 5%, and 10% levels of significance, respectively. The bold values mean regression coefficients*.

(2) Heterogeneity analysis based on enterprise industry type. On the one hand, green finance should support the development and growth of emerging green industries, and on the other hand, it should promote the green transformation and upgrading of traditional industries. Therefore, whether it is a new or traditional industry, green finance plays a supporting role to a certain extent. In this paper, the sample is divided into high-tech industry enterprises and traditional industry enterprises for grouping regression. The industry classification standard refers to China High-tech Industry Statistical Yearbook, and the corresponding regression results are shown in columns (3) and (4) of Table 7. The regression results show that the pilot policy of green finance has significantly promoted the value of both types of enterprises, which shows that green finance has played a supporting and promoting role for both high-tech enterprises and traditional enterprises. In addition, in comparison, the green finance pilot plays a more significant role in enhancing the value of enterprises in traditional industries, which indicates that the green finance pilot policy meets the needs of enterprises in traditional industries for green transformation and transformation and upgrading to a greater extent.
(3) Heterogeneity analysis based on enterprise size. Enterprises can be divided into large enterprises and small, medium, and micro-enterprises according to enterprise size. There are significant differences between them in enterprise size and various constraints. The corresponding regression results are shown in Columns (1) and (2) of Table 8. The regression results show that the green financial pilot policy can enhance the value of large enterprises and small and medium-sized enterprises in green enterprises. However, it has a more significant impact on large enterprises, while the regression results of small and medium-sized enterprises fail the significance test. The possible reason is that the existing green-finance-pilot policy design process did not fully consider solving the financing difficulties and expensive problems of small, medium, and micro-enterprises. The green finance policy plays a less prominent role in alleviating the financing constraints of small, medium, and micro enterprises, so it still mainly supported the development of large enterprises like traditional finance, thus enhancing the value of large green enterprises more obviously.
(4) Region-based heterogeneity analysis. Finally, this paper divides enterprises into the eastern, central, and western regions according to their registered addresses and discusses the impact of green finance pilot policies in different regions. The corresponding regression results are shown in columns (3–5) of Table 8. From the regression results, it can be seen that the green financial pilot has the most significant effect on the promotion of the value of green enterprises in the eastern region, while it has no apparent effect on the promotion of the value of green enterprises in the central region. For green enterprises in the western region, the impact of the green financial pilot is even harmful. The regression results show that from the point of view of promoting the value of the green enterprise, east green pilot financial policy effect is best, in the central and western regions is poorer, may cause of this result is that due to our country economy presents the apparent characteristics of plate ladder, east to the Midwest in the different stages of development, so China's relatively developed eastern region focus more on developing green transformation. However, the central and western regions pay more attention to the speed and scale of economic development, and green transformation is not in a priority position. Meanwhile, the eastern region is superior to the central and western regions in terms of infrastructure, financial development, and environmental law enforcement. Therefore, the green finance pilot can play a more significant role in the eastern region, that is, to significantly enhance the value of green enterprises, while the pilot policies in the central and western regions have little effect.

**Table 8:** Heterogeneity analysis results 2.

	**Large-scale enterprises**	**Small to micro enterprises**	**Eastern region**	**Central region**	**Western region**
	***q***	***q***	***q***	***q***	***q***
	**(1)**	**(2)**	**(3)**	**(4)**	**(5)**
*Treat*_*i*_×*Time*_*t*_	0.077** **(2.57)**	**0.063** **(0.84)**	0.100*** **(3.21)**	**0.081** **(0.69)**	**−0.044** **(−0.47)**
Control variables	Controlled	Controlled	Controlled	Controlled	Controlled
Entity fixed effects	Yes	Yes	Yes	Yes	Yes
Time fixed effects	Yes	Yes	Yes	Yes	Yes
*N*	5,187	1,578	4,456	1,200	1,109
*R* ^2^	0.379	0.427	0.454	0.302	0.294

***, **, * *represent the 1%, 5%, and 10% levels of significance, respectively. The bold values mean regression coefficients*.

## Conclusions

This paper takes the policy of green finance reform and innovation pilot zone in June 2017 as a quasi-natural experiment, and based on the relevant data of green enterprises in A-share listed companies; it examines the impact of green finance pilot policies on the long-term value of green enterprises in pilot areas by using double-difference method. The results show that the green finance pilot has significantly improved the enterprise value measured by Tobin *Q* in the long run and has a more significant impact on private enterprises, traditional industries, large enterprises, and green enterprises in the eastern region. At the same time, the greater the intensity of environmental regulation and the higher the level of financial development in a region, the more pronounced the effect of green finance pilot. The mechanism analysis shows that the green finance pilot policy mainly promotes the long-term value of green enterprises through the capital market effect of increasing the activity level of stock trading and the real effect of improving enterprise operation. The research results of this paper show that the policy of green finance reform and innovation pilot area promotes the development and value enhancement of green enterprises in the pilot area for a long time, and the regional pilot of green finance has achieved sound policy effects. The research results of this paper show that the development of green finance ultimately enhances the value of green enterprises by improving the capital market performance and actual operating performance of green enterprises, and provides a huge incentive for the green transformation and development of micro enterprises. Under the impact of the Covid-19, the above-mentioned effects of green finance will promote the green transformation of the economy at the micro-enterprise level, and then realize the green recovery of the economy.

Based on the research conclusions of this paper, policy implications can be obtained from the following three aspects: first, further, expand and promote the scope of green finance pilot to promote the development of green enterprises and green economic transformation. The research of this paper shows that the pilot policy of green finance can significantly enhance the long-term value of green enterprises in the pilot area and has a substantial policy effect. It is necessary to support the development and growth of green enterprises to promote the green transformation of the economy. The policy support of green finance is helpful to enhance the value and operational capability of green enterprises. Therefore, the next step should be to expand the coverage of green financial policies in industries and regions, further improve relevant institutional mechanisms, and alleviate the financing constraints faced by green enterprises. Especially in the post-epidemic era, vigorously developing green finance will help promote the green recovery of the economy.

Second, in developing green finance, attention shall be paid to the coordination of policies in other aspects. This study shows that the stronger the environmental regulation policy and the higher the level of financial development, the stronger the policy effect of the green financial pilot. Green finance has dual attributes of “environment” and “finance.” Therefore, when formulating policies and measures to support the development of green finance in the next step, on the one hand, environmental law enforcement should be strengthened to curb the development of highly polluting industries firmly; on the other hand, financial infrastructure should be improved to lay a solid foundation for the development of green finance, to play a synergistic effect and jointly promote the green transformation of the financial system.

Third, the formulation of green financial policies should fully consider the heterogeneity and regional characteristics of enterprises. The heterogeneity analysis of this paper shows that the pilot green finance has little impact on small and medium-sized micro-green enterprises and green enterprises in the central and western regions, indicating that the effect of the pilot green finance on alleviating the financing constraints of small and medium-sized micro-green enterprises is still not noticeable, and it has little effect on the central and western regions which are still in the underdeveloped stage. At present, it is more urgent to solve the requirements of financing difficulties and expensive financing for small, medium, and micro-enterprises, and the related problems are also urgent for green enterprises in small, medium, and micro-enterprises. Therefore, the next step in the design of green financial policies should be more inclined to support the green transformation and development of small, medium, and micro-enterprises and alleviate the financing constraints of the small, medium, and microgreen enterprises, to achieve a win-win policy of green transformation and to alleviate the problematic financing of small, medium and micro-enterprises. Because the central and western regions are relatively underdeveloped, they pay more attention to the speed and scale of economic development. The green transformation is not in a priority position, which ultimately makes the green financial pilot in the central and western regions have little effect.” lucid waters and lush mountains are invaluable assets,” therefore, in the next step, the assessment criteria should be improved. The weight of green development and environmental improvement in local development assessment indicators should be further enhanced. Meanwhile, efforts should be made to improve the financial development level and environmental law enforcement in the central and western regions to promote the green transformation of the central and western regions.

Finally, at present, for a developing country like China, to achieve green recovery, it is still facing real difficulties such as high green transition costs and insufficient technological innovation capabilities. Therefore, the next step should be to vigorously develop green finance as an important guarantee for green recovery. On the one hand, in terms of corporate investment and financing, China should formulate calibrated quantitative standards as soon as possible, form a complete green investment and financing standard system, and provide support and guidance for corporate green financing, so as to standardize the development model and direction of green industries. In response to the difficulties faced by enterprises in the process of green recovery, green finance should focus on supporting enterprises' technological upgrading and digital transformation. On the other hand, climate and environmental risk management is also a problem that needs to be addressed in the next step for green finance. Innovative green insurance and green financial derivatives markets can provide risk protection for the green and low-carbon transformation of enterprises, thereby ensuring a stable economic recovery in the post-epidemic era.

## Data Availability Statement

The datasets presented in this study can be found in online repositories. The names of the repository/repositories and accession number(s) can be found at: https://www.gtarsc.com/; https://www.wind.com.cn/NewSite/edb.html.

## Author Contributions

JH, JL, YL, WW, and LZ: conceptualization. XL and WW: methodology. JH, JL, and WW: formal analysis and investigation. YL and LZ: supervision. XL: validation. All authors contributed to the article and approved the submitted version.

## Funding

This work was supported by the National Natural Science Foundation of China Youth Project (71902050).

## Conflict of Interest

The authors declare that the research was conducted in the absence of any commercial or financial relationships that could be construed as a potential conflict of interest.

## Publisher's Note

All claims expressed in this article are solely those of the authors and do not necessarily represent those of their affiliated organizations, or those of the publisher, the editors and the reviewers. Any product that may be evaluated in this article, or claim that may be made by its manufacturer, is not guaranteed or endorsed by the publisher.
